# Image-Guided Robotic Radiosurgery for the Management of Spinal Ependymomas

**DOI:** 10.3389/fonc.2021.654251

**Published:** 2021-04-29

**Authors:** Felix Ehret, Markus Kufeld, Christoph Fürweger, Alfred Haidenberger, Paul Windisch, Carolin Senger, Melina Kord, Malte Träger, David Kaul, Christian Schichor, Jörg-Christian Tonn, Alexander Muacevic

**Affiliations:** ^1^ Charité – Universitätsmedizin Berlin, Corporate Member of Freie Universität Berlin and Humboldt-Universität zu Berlin, Department of Radiation Oncology, Berlin, Germany; ^2^ European Cyberknife Center, Munich, Germany; ^3^ Department of Stereotaxy and Functional Neurosurgery, University Hospital Cologne, Cologne, Germany; ^4^ Department of Radiation Oncology, Kantonsspital Winterthur, Winterthur, Switzerland; ^5^ Charité – Universitätsmedizin Berlin, Corporate Member of Freie Universität Berlin and Humboldt-Universität zu Berlin, Charité CyberKnife Center, Berlin, Germany; ^6^ German Cancer Consortium (DKTK), Partner Site Berlin, German Cancer Research Center (DKFZ), Heidelberg, Germany; ^7^ Department of Neurosurgery, Ludwig-Maximilians-University Munich, Munich, Germany

**Keywords:** ependymoma, ependymal tumors, radiosurgery, SBRT, spine, CyberKnife

## Abstract

**Background:**

Ependymomas are rare neoplasms of the central nervous system (CNS), usually localized intracranially and most commonly diagnosed in children. Spinal ependymomas are more frequent in young adults. They are either primary lesions or manifest as disseminated seeding of cranial tumors. Data on the management of spinal ependymoma lesions remain scarce, especially concerning stereotactic radiosurgery (SRS) and stereotactic body radiation therapy (SBRT). The purpose of this study is to report the treatment outcomes of two institutions using robotic radiosurgery (RRS) for the treatment of spinal ependymomas.

**Materials and Methods:**

All patients with a histopathologically confirmed diagnosis of an ependymoma WHO grade II or III who were treated with RRS for one or more spinal lesions were included in this analysis.

**Results:**

Twelve patients underwent RRS for the treatment of 32 spinal ependymoma lesions between 2005 and 2020. Two patients were below the age of 18 when treated, whereas nine patients (75%) suffered from a primary spinal ependymoma. The median dose was 15 Gy prescribed to a median isodose of 70%, with 27 lesions (84%) receiving a single-session treatment. The local control (LC) after a median follow-up of 56.7 months was 84%. LC rates at 1, 3, and 5 years were 92, 85, and 77%, respectively. The Kaplan-Meier estimated overall survival after 1, 3, and 5 years were 75, 75, and 64%, respectively. Five patients died, all of them suffering from an anaplastic ependymoma, with widespread CNS tumor progression being the reason for death in four patients. The majority of patients (58%) showed a stable neurological status at the last available follow-up. Overall, the treatment was well tolerated.

**Conclusion:**

RRS appears to be a safe and efficient treatment modality for managing primary and secondary spinal ependymal tumors in patients with multiple lesions and local recurrences.

## Introduction

Ependymomas or ependymal tumors are rare neoplasms of the central nervous system (CNS) and of neuroectodermal origin (1, 2). With an estimated annual incidence of 0.43 per 100.000 and year, this tumor entity accounts for 1.7% of all primary CNS tumors (3). Ependymomas are more commonly found in children and young adults, where they represent 4.7% of all CNS tumors (3). These tumors arise from the ependymal lining of the cerebral ventricles, choroid plexus, and central canal of the spinal cord. Locally distinct radial glia cells of the subventricular zone are supposed to be the cells of origin of ependymoma. Spinal ependymomas are more commonly found in young adults, whereas most of the ependymal tumors in children are intracranially located (3–5). Today, nine distinct molecular subgroups based on DNA methylation patterns have been identified, which may guide and advance personalized therapies (1, 6, 7). Today, the mainstay of treatment is the gross total surgical tumor resection as recommended by the European Association of Neuro-Oncology (EANO) (1). Depending on the World Health Organization (WHO) grading, location, and extent of surgical resection, adjuvant radiotherapy and chemotherapy are further components of the multimodal treatment (1, 8). In patients with poor Karnofsky Performance Status (KPS), tumor relapses, widespread disease, and multiple spinal lesions, surgery and conventional radiotherapy may not be repeatable or feasible (1, 2). Stereotactic radiosurgery (SRS) and stereotactic body radiation therapy (SBRT) may be salvage treatment options and help to stop local tumor progression - at least temporarily (9–11). Information on the treatment outcomes after SRS and SBRT for ependymal tumor lesions are scarce, especially concerning spinal ependymomas and robotic radiosurgery (RRS) (10, 12, 13). To the best of our knowledge, only two other reports dedicated to spinal ependymomas are available in the English literature to date (10, 13). Herein, we report the 15-year institutional experience of two treatment centers, including results on local tumor control, treatment characteristics, survival outcomes, and adverse events (AE).

## Materials and Methods

All patients who were treated at two institutions for a spinal ependymal tumor between 2005 and 2020 were included in this retrospective analysis. Only patients with a histopathological ependymoma diagnosis, including grading according to the WHO CNS tumor classification, were eligible. Indication for RRS was confirmed by an interdisciplinary neurooncological tumor board involving neurosurgeons, neuroradiologists, neuropathologists, and radiation oncologists. Medical history, including pretreatments, treatment plans, neurological deficits, imaging data, and histology, were either stored in a dedicated radiosurgical database or hospital records (14). All patients underwent image-guided RRS using a CyberKnife® robotic radiosurgery system (Accuray Inc., Sunnyvale, CA, USA). Patients only undergoing biopsy for histological confirmation were classified as non-surgical cases. For treatment delivery, contrast-enhanced computed tomography (CT) and magnetic resonance imaging (MRI) scans were acquired for every patient and subsequently overlaid for inverse treatment planning with changing versions of proprietary planning software (MultiPlan, Precision, Accuray Inc., Sunnyvale, CA, USA). Local tumor response, clinical symptoms, and AE were evaluated clinically and by MRI assessment every three months for the first year, then every six months during follow-up or depending on the patients’ status and clinical suspicion for tumor progression. The Kaplan-Meier estimate was applied for the respective analyses on the length of local control (LC) and overall survival (OS). LC was defined as an unchanged or decreased tumor volume on follow-up imaging, whereas local failure was defined as an increased tumor volume during follow-up. AE were assessed by the clinical notes of the respective physician and available imaging data. Data were tested for normal distribution by the Shapiro-Wilk test and graphical appearance, including skewness and kurtosis. Normally distributed continuous variables were analyzed with the unpaired student’s t-test, non-normally distributed data with the Wilcoxon rank-sum test. All p-values were two-sided and statistical significance was defined as p<0.05. Statistical analyses were performed with STATA MP 16.0 (StataCorp, College Station, TX, USA). This study was approved by the institutional review board of the Ludwig-Maximilians-University Munich (20-256 KB).

## Results

### Patients and Treatment Characteristics

Twelve patients with 32 spinal ependymoma lesions between 2005 and 2020 were included in this analysis. The majority of patients were male (67%). Two patients (17%) were below the age of 18 when treated, with a median age of 34.9 years at the time of RRS. Before treatment, four (33%) patients did not show any symptoms, whereas the remaining eight suffered mostly from motoric weakness or paralysis (58%) and sensory deficits (41%). Most of the treated lesions were located in the thoracic spine (56%), with RRS being the primary treatment modality for the majority of all lesions (59%). Eight treated lesions (25%) were local recurrences, and five (16%) received adjuvant RRS after incomplete surgical resection. Before RRS, four patients had received multiple cycles of systemic treatments. No patients received chemotherapy during RRS treatment. Nine patients (75%) suffered from a primary spinal ependymoma, whereas the remaining three had a primary ependymal intracranial tumor before developing spinal tumor lesions. The intracranial tumors were initially located in the fourth ventricle, the cerebellum, and the parietal as well as occipital lobes. All patients underwent at least one biopsy or surgical resection of a tumor lesion for histopathological examination. According to the WHO classification of CNS tumors, seven ependymomas (66%) were grade II, with the remaining five being classified as anaplastic ependymomas (grade III). No patient suffered from neurofibromatosis. The median KPS before RRS was 80%, ranging from 30 to 100%. The overall median prescription dose was 15 Gray (Gy), either delivered in a single fraction (range 10 to 16.5 Gy) or three fractions (range 21 to 24 Gy). WHO grade III tumors received doses ranging from 10 to 24 Gy, whereas grade II tumors received doses between 14 and 16.5 Gy. The median prescription isodose was 70%. Twenty-seven of the 32 lesions (84%) were treated with one fraction, the remaining five lesions (16%) of two patients received three fractions. The median irradiated tumor volume was 0.37 cc, ranging from 0.03 cc in a primarily resected lesion to 2.89 cc in an unresected tumor lesion. The patient and treatment characteristics are summarized in **Table 1**.

**Table 1:** Patient and treatment characteristics.

Number of patients	12				
Number of lesions	32				
Gender (male/female)	8		4		
%	67		33		
	**Median**	**Mean**			**Range**
Age (years)	34.9	35.1			13.7 – 71.3
Pretreatment Karnofsky Performance Status (%)	80	80			30 – 100
Follow-up (months)	56.7	55.7			3.2 – 104.2
Tumor volume (cc)	0.37	0.58			0.03 – 2.89
Number of fractions	1	1.3			1 – 3
Dose (Gy)	15	15.6			10 – 24
Prescription isodose (%)	70	71.8			70 – 85
Conformity index	1.32	1.43			1.04 – 2.67
Homogeneity index	1.43	1.39			1.18 – 1.43
Coverage	97.3	93.7			76.0 – 99.9
RRS indication	**Primary treatment**	**Recurrence**			**Adjuvant treatment**
Number of lesions	19	8			5
%	59	25			16
Tumor location	**Cervical**	**Thoracic**			**Lumbar**
Number of lesions	10	18			4
%	31	56			13
Tumor grading (WHO)	**II**			**III**	
Number of patients	7			5	
%	58			42	
Symptoms	**None**	**Dysesthesia/hypesthesia**			**Weakness/paralysis**
Number of patients	4	5			7
%	33	41			58

cc, cubic centimeter; Gy, Gray; WHO, World Health Organization.

### Local Outcome and Survival Data

**Figure 1:**
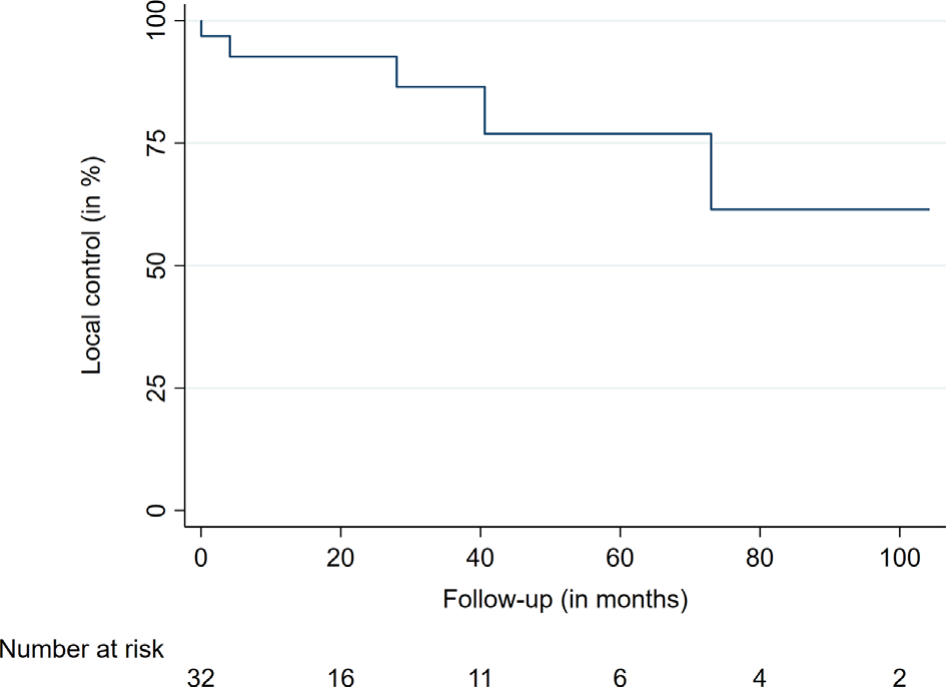
Local control.

**Figure 2:**
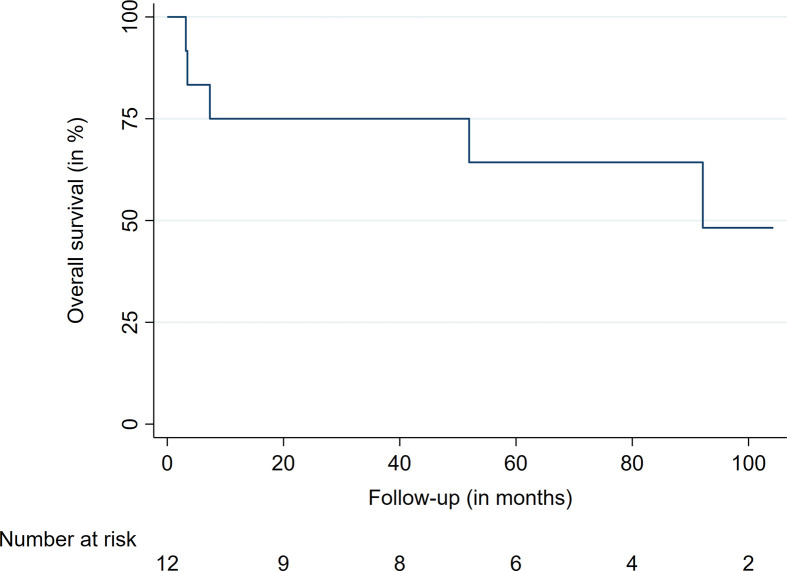
Overall survival.

The median follow-up time was 56.7 months, ranging from 3.2 to 104.2 months. At the last available follow-up, 27 of the 32 treated lesions were controlled, leading to a local control (LC) rate of 84%. The LC rates after 12, 36, and 60 months were 92%, 85%, and 77%, respectively (**Table 2**, **Figure 1**). The median time to local failure was 28 months. The five local recurrences after RRS occurred in four patients, with three (75%) of them suffering from an anaplastic ependymoma. No significant differences were observed between locally controlled and uncontrolled lesions in regard to dose, prescription isodose, fractions, or tumor volume due to the limited number of events. At the last available follow-up, five (42%) patients had died after a median time of seven months. All were suffering from an anaplastic ependymoma, with widespread CNS tumor progression being the reason for death in four patients. Three of these four tumor-associated deaths occurred in male patients. One patient died from tumor-unrelated causes. The overall survival rates at 12, 36, and 60 months were 75%, 75%, and 64%, respectively (**Table 2**, **Figure 2**). Of the four patients who had not shown symptoms before treatment, two remained stable throughout the follow-up, with the remaining two experiencing onsets of new symptoms (unsteady gait and back pain). The remaining eight patients mostly presented with an unchanged neurological status (5 patients, 62%), with two patients (25%) showing progressing clinical deficits. One patient (12%) who suffered from ataxia fully recovered after treatment and remained symptom-free. The treatment was well tolerated in the majority of patients. One patient who was treated for four lesions in the thoracic spine developed edema at the treatment sites shortly after RRS. He was successfully treated with glucocorticoids. No treatment-related deaths, bleedings, radiation necrosis, or radiation-induced malignancies have been observed.

**Table 2:** Local control and overall survival data.

Variable	Time (in months)	%
LC	12	92.8
	24	92.8
	36	85.9
	48	77.3
	60	77.3
OS	12	75.0
	24	75.0
	36	75.0
	48	64.2
	60	64.2

LC, local control; OS, overall survival.

## Discussion

To the best of our knowledge, we report the most extensive series of radiosurgically treated spinal ependymal tumors. With most of the available SRS and SBRT data for ependymomas focusing on intracranial lesions, outcome data for spinal treatments remain sparse (10–12, 15–18). Moreover, the majority of previous studies on SRS utilized GammaKnife (GK)- or conventional linear accelerator (LINAC)-based radiosurgery (11, 12, 15–17, 19–21). Previous studies analyzing SRS and SBRT for intracranial and spinal ependymal tumors observed LC rates typically ranging between 60 and 80% (10, 13, 15, 16, 19–22). It is important to note that previous and the current study populations are heterogeneous, especially concerning age, tumor location, and previous treatments, including the degree of upfront surgical resection. This limits the comparability of the current study and past analyses. Nevertheless, our observed LC of 84% at the last available follow-up is a plausible finding. Shi et al. and Ryu et al. both analyzed cases treated at Stanford University and reported on the radiosurgical treatment of spinal ependymal lesions (10, 13). A total of 13 spinal lesions in 9 patients were treated with RRS in both studies, leading to a LC of 92% (10, 13). Notably, both studies had a shorter median follow-up and sample size than in the current series, which may account for the better results besides varying upfront treatments and the inclusion of intracranial treatments (12 and 54 months vs. 56.7 months) (10, 13). Both studies showed a favorable risk profile (10, 13). Our treatments were also well tolerated, and no severe AE were observed throughout the available follow-up.

Concerning the clinical outcomes, Ryu et al. reported improvement of the two treated patients (13). In contrast, Shi et al. did not report clinical outcomes after treatment (10). Herein, we observed stabilization of pretreatment deficits in most patients. Yet, four patients had either progressive symptoms (two) or developed new neurological deficits (two). Together with our results, RRS appears to be an effective and safe local treatment modality to limit spinal tumor progression and further neurological decline in most cases. Despite the limited sample size and patient heterogeneity, these findings may help to delay or avoid craniospinal irradiation (CSI) or repeated fractionated radiotherapy and associated AE in selected patients (23–26). In regard to the general management of intracranial and spinal ependymoma patients, the EANO have published its recommendations and guidelines in 2017 (1). In case of the spinal ependymoma recommendations, the use of SRS or SBRT is not endorsed and remains unclear, most likely due to the lack of available data and studies (1, 12). On the other hand, fractionated radiotherapy is currently recommended as an adjuvant treatment modality after complete (WHO grade III) and incomplete (WHO II and III) surgical resection in this patient subgroup, with doses ranging from 54 to 59.4 Gy (1). With the increasing availability of SRS and RRS, the number of spinal ependymal treatments may increase and help to clarify its role in the management of this challenging patient group.

Survival of patients with ependymal tumors seems to be mainly dependent on DNA methylation profiles, the extent of surgical resection, and 1q gain (6, 7). Given the recent identification of these genetic parameters and the rarity of the tumor, large prospective validations are lacking (1, 6, 7). Previous studies identified differing predictors of OS and progression-free survival (PFS), including the extent of first surgical resection, WHO grade III, intracranial tumor location as well as age, gender and tumor volume at the time of reirradiation (11, 27, 28). Our study cohort mainly consisted of adult patients (83%), with most of them suffering from a primary spinal ependymoma (75%). This subgroup of patients is known to have a more favorable outcome compared to pediatric patients with intracranial tumors (6, 7, 28). However, all four patients who succumbed to their ependymal tumors in this series were diagnosed with an anaplastic ependymoma (WHO grade III), with two of them suffering from a primary spinal ependymal tumor. Despite the small sample size, one may conclude that anaplastic histopathological features have an impact on OS. This finding is in agreement with previous studies (27, 28). However, DNA methylation data are lacking in our patients, limiting comparability and risk stratification beyond the WHO classification. Further limitations of this study include the retrospective nature, patient heterogeneity, small sample size, and a potential sampling bias. All these factors may limit the drawn conclusions of this study. Nevertheless, this study provides more evidence on the efficacy and safety of RRS for spinal ependymomas.

## Conclusion

Spinal ependymal tumors may be efficiently treated with RRS, especially in patients with multiple lesions and local recurrences after surgical resection and adjuvant radiotherapy. Most lesions remained controlled, and the treatment was well tolerated. Further neurological decline was prevented in the majority of patients. RRS may be a preferable, time-saving, and non-invasive treatment modality for selected patients.

## Data Availability Statement

The data that support the findings of this study are available from the corresponding author, FE, upon reasonable request. 

## Ethics Statement

The studies involving human participants were reviewed and approved by the Institutional Review Boards of the Ludwig-Maximilians-University Munich and Charité – Universitätsmedizin Berlin. Written informed consent for participation was not required for this study in accordance with the national legislation and the institutional requirements.

## Author Contributions

Conception and design of the study: FE. Data acquisition: FE, MKu, CF, AH, CSe, MKo, AM. Data analysis and drafting of the manuscript: FE. Critically revising manuscript: FE, MKu, CF, PW, CSe, MT, DK, CSc, J-CT, AM. All authors contributed to the article and approved the submitted version.

## Conflict of Interest

FE reports a grant from Ludwig-Maximilians-University Munich and honoraria from Accuray outside the submitted work.

The remaining authors declare that the research was conducted in the absence of any commercial or financial relationships that could be construed as a potential conflict of interest.
